# Seafood Consumption, Omega-3 Fatty Acids Intake, and Life-Time Prevalence of Depression in the PREDIMED-Plus Trial

**DOI:** 10.3390/nu10122000

**Published:** 2018-12-18

**Authors:** Almudena Sánchez-Villegas, Jacqueline Álvarez-Pérez, Estefanía Toledo, Jordi Salas-Salvadó, Carolina Ortega-Azorín, Maria Dolores Zomeño, Jesús Vioque, Jose Alfredo Martínez, Dora Romaguera, Jessica Pérez-López, José López-Miranda, Ramón Estruch, Aurora Bueno-Cavanillas, Fernando Arós, Josep A. Tur, Francisco J. Tinahones, Oscar Lecea, Vicente Martín, M. Ortega-Calvo, Clotilde Vázquez, Xavier Pintó, Josep Vidal, Lidia Daimiel, Miguel Delgado-Rodríguez, Pilar Matía, Dolores Corella, Andrés Díaz-López, Nancy Babio, Miguel Ángel Muñoz, Montserrat Fitó, Manoli García de la Hera, Itziar Abete, Antonio García-Rios, Emilio Ros, Miguel Ruíz-Canela, Miguel Ángel Martínez-González, Marisol Izquierdo, Lluis Serra-Majem

**Affiliations:** 1 Nutrition Research Group, Research Institute of Biomedical and Health Sciences, University of Las Palmas de Gran Canaria, 35016 Las Palmas de Gran Canaria, Spain; jackyalvarez62@gmail.com (J.A.-P.); lluis.serra@ulpgc.es (L.S.-M.); 2 Consorcio CIBER, M.P. Fisiopatología de la Obesidad y Nutrición (CIBERObn), Instituto de Salud Carlos III (ISCIII), 28029 Madrid, Spain; etoledo@unav.es (E.T.); jordi.salas@urv.cat (J.S.-S.); carolina.ortega@uv.es (C.O.-A.); jalfmtz@unav.es (J.A.M.); d.romaguera-bosch@imperial.ac.uk (D.R.); jlopezmir@gmail.com (J.L.-M.); restruch@clinic.cat (R.E.); aborau@secardiologia.es (F.A.); pep.tur@uib.es (J.A.T.); fjtinahones@uma.es (F.J.T.); oscar.lecea.juarez@cfnavarra.es (O.L.); mortega7@us.es (M.O.-C.); cvazquezma@gmail.com (C.V.); xpinto@bellvitgehospital.cat (X.P.); dolores.corella@uv.es (D.C.); andres.diaz@urv.cat (A.D.-L.); nancy.babio@urv.cat (N.B.); mfito@imim.es (M.F.); angarios2004@yahoo.es (A.G.-R.); eros@clinic.cat (E.R.); mcanela@unav.es (M.R.-C.); mamartinez@unav.es (M.A.M.-G.); 3 Department of Preventive Medicine and Public Health, University of Navarra-IdiSNA, 31008 Pamplona, Spain; 4 Department of Biochemistry and Biotechnology, Human Nutrition Unit, Universitat Rovira i Virgili, 43204 Reus, Spain; 5 Institut d’Investigació Sanitària Pere Virgili (IISPV), 43204 Reus, Spain; 6 Department of Internal Medicine. University Hospital of Sant Joan de Reus, 43204 Reus, Spain; 7 Department of Preventive Medicine, University of Valencia, 46010 Valencia, Spain; 8 Cardiovascular Risk and Nutrition, IMIM-Hospital del Mar Medical Research Institute, 08003 Barcelona, Spain; mariadoloreszf@blanquerna.edu; 9 Blanquerna School of Life Sciences, Universitat Ramon Llull, 08025 Barcelona, Spain; 10 Consorcio CIBER, M.P. Epidemiología y Salud Pública (CIBEREsp), Instituto de Salud Carlos III (ISCIII), 28029 Madrid, Spain; vioque@umh.es (J.V.); abueno@ugr.es (A.B.-C.); vicente.martin@unileon.es (V.M.); mdelgado@ujaen.es (M.D.-R.); manoli@umh.es (M.G.d.l.H.); 11 Nutritional Epidemiology Unit, Miguel Hernández University, ISABIAL-FISABIO, 46020 Alicante, Spain; 12 Department of Nutrition and Food Sciences, Physiology and Toxicology, University of Navarra, 31008 Pamplona, Spain; iabetego@unav.es; 13 IMDEA Food, CEI UAM + CSIC, 28049 Madrid, Spain; lidia.daimiel@imdea.org; 14 Health Research Institute of the Balearic Islands (IdISBa), University Hospital Son Espases, 07120 Palma de Mallorca, Spain; 15 School of Health Sciences. University of Málaga-IBIMA, 29010 Malaga, Spain; jwarnberg@uma.es; 16 Department of Internal Medicine, Reina Sofia University Hospital, University of Córdoba-IMIBIC, 14004 Córdoba, Spain; 17 Department of Internal Medicine, IDIBAPS, Hospital Clinic, University of Barcelona, 08036 Barcelona, Spain; 18 Department of Preventive Medicine, University of Granada, 18016 Granada, Spain; 19 Department of Cardiology OSI ARABA, University Hospital Araba, 01009 Vitoria, Spain; 20 Department of Medicine, University of the Basque Country UPV/EHU, 01006 Vitoria-Gasteiz, Spain; 21 Research Group on Community Nutrition and Oxidative Stress, Universitat de les Illes Balears, 07122 Palma de Mallorca, Spain; 22 Department of Endocrinology, University Hospital, University of Málaga, 29010 Málaga, Spain; 23 Atención Primaria, Osasunbidea-Servicio Navarro de Salud, 31002 Pamplona, Spain; 24 Instituto de Biomedicina (IBIOMED), Universidad de León, 24071 León, Spain; 25 Department of Family Medicine, Distrito Sanitario Atencion Primaria, Centro de Salud Las Palmeritas, 41005 Sevilla, Spain; 26 Department of Endocrinology, Fundación Jiménez-Díaz, 28040 Madrid, Spain; 27 Lipids and Vascular Risk Unit, Internal Medicine, Hospital Universitario de Bellvitge, Hospitalet de Llobregat, 08907 Barcelona, Spain; 28 Consorcio CIBER, M.P. Diabetes y enfermedades metabólicas (CIBERDem), Instituto de Salud Carlos III (ISCIII), 28029 Madrid, Spain; jovidal@clinic.cat; 29 Department of Endocrinology, IDIBAPS, Hospital Clinic, University of Barcelona, 08036 Barcelona, Spain; 30 Division of Preventive Medicine, University of Jaén, 23071 Jaén, Spain; 31 Instituto de Investigación Sanitaria del Hospital Clínico San Carlos, 28040 Madrid, Spain; pilar.matia@gmail.com; 32 Lipids and Cardiovascular Epidemiology Research Unit, Institut Municipal d’Investigació Mèdica (IMIM), 08003 Barcelona, Spain; 33 Primary Health Care Division. Institut Català de la Salut and IDIAPJgol, 08007 Barcelona, Spain; mamunoz.bcn.ics@gencat.cat; 34 Department of Nutrition, Harvard T. H. Chan School of Public Health, Boston, MA 02115, USA; 35 Ecoaqua Institute, University of Las Palmas de Gran Canaria, 35214 Telde, Spain; marisol.izquierdo@ulpgc.es

**Keywords:** fish, omega-3, eicosapentaenoic acid, docosahexaenoic acid, depression

## Abstract

Background: The aim of this analysis was to ascertain the type of relationship between fish and seafood consumption, omega-3 polyunsaturated fatty acids (ω-3 PUFA) intake, and depression prevalence. Methods: Cross-sectional analyses of the PREDIMED-Plus trial. Fish and seafood consumption and ω-3 PUFA intake were assessed through a validated food-frequency questionnaire. Self-reported life-time medical diagnosis of depression or use of antidepressants was considered as outcome. Depressive symptoms were collected by the Beck Depression Inventory-II. Logistic regression models were used to estimate the association between seafood products and ω-3 PUFA consumption and depression. Multiple linear regression models were fitted to assess the association between fish and long-chain (LC) ω-3 PUFA intake and depressive symptoms. Results: Out of 6587 participants, there were 1367 cases of depression. Total seafood consumption was not associated with depression. The odds ratios (ORs) (95% confidence intervals (CIs)) for the 2nd, 3rd, and 4th quintiles of consumption of fatty fish were 0.77 (0.63–0.94), 0.71 (0.58–0.87), and 0.78 (0.64–0.96), respectively, and *p* for trend = 0.759. Moderate intake of total LC ω-3 PUFA (approximately 0.5–1 g/day) was significantly associated with a lower prevalence of depression. Conclusion: In our study, moderate fish and LC ω-3 PUFA intake, but not high intake, was associated with lower odds of depression suggesting a U-shaped relationship.

## 1. Introduction

Unipolar depression is identified as one of the leading causes of burden of disease worldwide, measured in adjusted life years [1]. In addition to the profound effects on the functional capacity and quality of life of affected individuals, it has a major impact on mortality risk by suicide, cardiovascular disease, and other diseases as well as death by all causes [2].

According to the International Society for Nutritional Psychiatry Research, although the growth in scientific research related to nutrition in psychiatry may be recent, it is now at a stage where it can no longer be ignored [3]. In this sense, the beneficial effects of long-chain omega-3 polyunsaturated fatty acid intake (LC ω-3 PUFA), mainly eicosapentaenoic acid (EPA) and docosahexaenoic acid (DHA), on the adequate nervous system function is largely known [4]. The anti-inflammatory capacity of LC ω-3 PUFA, particularly EPA, could be crucial to prevent depression development. In fact, up to 50% of depressed participants have higher level of pro-inflammatory cytokines that are implicated in hypothalamic–pituitary–adrenal axis regulation, neurotransmitter metabolism, or even in the composition of intestinal microbiota [5]. Several meta-analyses have evaluated the beneficial role of LC ω-3 PUFA supplementation in several mental disorders such as bipolar disorder [6], schizophrenia [7], or depression [8]. In this sense, EPA supplementation has been proposed as an important coadjuvant of antidepressant treatment [9]. Moreover, several epidemiological studies have also reported an inverse relationship between LC ω-3 PUFA intake and depression prevalence and incidence. In a recent meta-analysis, total ω-3 PUFA as well as LC ω-3 PUFA intake were associated with significant relative risk (RR) reductions: RR for the highest versus lowest intake category= 0.82 (95% confidence interval (CI) = 0,72–0.94) and RR = 0.76 (95% CI = 0.64–0.90), respectively [10]. However, the results are not consistent across studies. In other recent meta-analysis, Yang et al. did not find a significant association between ω-3 PUFA intake and depression: RR for the highest versus lowest category of intake = 0.87 (95% CI = 0.74–1.04) [11]. This study also failed to find a dose–response relationship between ω-3 PUFA intake and depression [11]. One possible explanation for these inconsistent results could be the potential existence of a non-linear threshold effect for ω-3 PUFA intake [10] or the existence of a differential effect of ω-3 PUFA intake according to the sex of the participants as some studies suggest [12,13]. Recent evidence also supports an inverse association between fish consumption and depression that could differ by sex [14,15,16,17]. Most of these observational studies have found a significant reduction in the risk of depression associated with fish or ω-3 PUFA intake particularly in females [12,14,16,17].

The aims of this study were (1) to cross-sectionally analyze the association between the consumption of different types of seafood products and ω-3 PUFA and depression, (2) to establish the shape of the dose–response curve and the potential existence of a non-linear threshold effect for ω-3 PUFA and, finally, (3) to ascertain if these associations differed by sex, the presence of cardio-metabolic disorders, or several life-style habits in the PREDIMED-Plus trial.

## 2. Methods

### 2.1. Design and Participants

This study was based on the cross-sectional analysis of baseline data within the frame of the PREDIMED-Plus trial, a six-year ongoing multicenter, randomized, parallel-group clinical trial conducted in Spain to assess the effect of an intensive weight-loss intervention based on an energy-restricted traditional Mediterranean diet, physical activity promotion, and behavioral support on hard cardiovascular events, in comparison with a control group receiving usual care intervention only with energy-unrestricted Mediterranean diet recommendations. A more detailed description of the PREDIMED-Plus study is available at http://predimedplus.com/. This study was registered at the International Standard Randomized Controlled Trial (ISRCT; http://www.isrctn.com/ISRCTN89898870) with number 89898870 (registration date: 24 July 2014).

A total of 6874 participants were recruited and randomized in 23 recruitment sites from different universities, hospitals, and research institutes of Spain. The eligible participants were community-dwelling adults (aged 55–75 in men; 60–75 in women) with overweight/obesity [body mass index (BMI) ≥ 27 and < 40 kg/m^2^], who met at least three components of the metabolic syndrome (MetS) according to the updated harmonized criteria of the International Diabetes Federation and the American Heart Association and National Heart, Lung, and Blood Institute [18].

For the present analysis, participants who were outside of predefined limits for baseline total energy intake (less than 800 Kcal/day or more than 4,000 Kcal/day in men, and less than 500 Kcal/day or more than 3,500 Kcal/day in women) (*n* = 259), and participants with missing data in smoking status (*n* = 28) were excluded from the analyses. Finally, 6587 participants were analyzed.

### 2.2. Exposure Assessment

Dietary intake was assessed with a validated 143-item semi-quantitative food-frequency questionnaire [19] administered at baseline. In face-to-face interviews, participants were asked about the frequency of consumption of each food item during the past year, specifying usual portion sizes. Nine possibilities of frequency were offered, ranging from never to > 6 times/day. Information on seafood products was collected for eight items of the food-frequency questionnaire (uncanned fatty fish; lean fish; smoked/salted fish; mollusks; shrimp, prawn, and crayfish; octopus, baby squid, and squid; fatty fish canned in oil; fatty fish canned in salted water). For this analysis, both canned fish in oil or in salted water were considered in the same category. Moreover, mollusks; shrimp, prawn, and crayfish; octopus, baby squid, and squid were labeled as other seafood. Quantities of EPA, DHA, and docosapentaenoic acid (DPA) were derived from these eight items.

Major sources of α-Linolenic acid (ALA) acid were collected through six items (soybean oil, walnuts, margarine, corn oil, sunflower oil, olive oil). Consumption of flaxseed and canola oils was not considered because these oils are not consumed in Spain. Nutrient intakes were computed using Spanish food composition tables [20,21]. The validation of the food-frequency questionnaire against four 3-day food records showed energy-adjusted intra-class correlation coefficients of 0.506 and 0.728 for LC ω-3 PUFA and ALA, respectively [19]. The intake of seafood products and ω-3 PUFA was adjusted for total energy intake using the residual method. Finally, energy-adjusted intake of seafood products, LC ω-3 PUFA, and ALA was categorized into quintiles.

### 2.3. Outcome Assessment

Depression was collected at baseline and was defined as a self-reported life-time medical diagnosis of depression or the habitual use of antidepressants by the participant. The use of self-reported medical diagnosis of depression collected through a questionnaire has been validated in another Spanish study showing adequate validity [22]. Moreover, depressive symptoms were also assessed at baseline through the Beck Depression Inventory-II validated in Spain. The Beck Depression Inventory-II includes 21 questions with four possible answers sorted according to symptom severity and score ranges from 0 to 63 points [23].

### 2.4. Covariate Assessment

Information about socio-demographic (e.g., sex, age, marital status, and educational level), and lifestyle-related variables (e.g., smoking status, physical activity, or adherence to the Mediterranean diet) were obtained from the baseline questionnaire.

Anthropometric variables were determined by trained staff and in accordance with the PREDIMED-Plus operations protocol. Weight and height were measured with calibrated scales and a wall-mounted stadiometer, respectively. BMI was calculated as the weight in kilograms divided by the height in meters squared.

Leisure-time physical activity was assessed using the short form of the Minnesota Leisure Time Physical Activity Questionnaire validated in Spain [24,25] (including questions to collect information about types of physical activity, their frequency (number of days), and duration (min/day)). Leisure-time activities were computed by assigning a metabolic equivalent score to each activity, multiplied by the time spent for each activity and summing up all activities. The intensity was assigned based on the compendium of physical activity [26].

The adherence to the Mediterranean diet was evaluated through the Mediterranean Diet Score (MDS) proposed by Trichopoulou and was based on the consumption of eight items (we excluded fish consumption from the original score based on nine elements: fruits and nuts, vegetables, legumes, cereals, ratio monounsaturated/saturated fatty acids, moderate alcohol consumption, fish, dairy products, and meat and meat products) [27]. Personal history of illness (hypertension, dyslipidemia, and type 2 diabetes) was collected from the patients’ medical records.

### 2.5. Statistical Analysis

Logistic regression models were fitted to assess the relationship between the energy-adjusted consumption of different types of fish and seafood products and intake of ω-3 PUFA (in quintiles) and the prevalence of depression. Odds ratios (ORs) and their 95% CIs were calculated considering the lowest quintile as the reference category. To control for potential confounding factors, the results were adjusted for sex, age, marital status (married/other), educational level (primary, secondary, university, unknown), smoking (non-smoker, former smoker, current smoker), physical activity during leisure time (quintiles of METs/min-w), BMI (kg/m^2^, continuous), presence of several diseases (hypertension, dyslipidemia, and type 2 diabetes) at baseline, and total energy intake (kcal/day, continuous) and adherence to the Mediterranean Diet (0–8, continuous). Tests of linear trend across increasing quintiles of exposures were conducted by assigning the medians to each quintile and treating them as continuous variables.

Moreover, the potential non-parametrical non-linear association between fatty fish consumption and LC ω-3 intake and prevalent depression was calculated with restricted cubic splines. Tests for non-linearity used the likelihood ratio test, comparing the model with only the linear term to the model with the linear and the cubic spline terms. The results were adjusted for the same potential confounding factors as the main logistic regression analysis. Multiple linear regression models were fitted to assess the association between the energy-adjusted consumption of different types of fish or LC ω-3 PUFA (in quintiles) and depressive symptoms assessed through the Beck Depression Inventory-II.

Finally, in order to assess the possible effect modification by sex, type 2 diabetes prevalence, adherence to the MDS, or smoking, product-terms were introduced in the different multivariable models. In addition, *p*-values for the interaction were calculated with the log-likelihood ratio test.

## 3. Results

We identified 1367 participants at baseline with life-time prevalence of depression. Table 1 shows the distribution of baseline characteristics of participants in the PREDIMED-Plus according to seafood product and LC ω-3 PUFA consumption. Participants with the highest consumption of seafood products and LC ω-3 PUFA were more likely to be women, who were never smokers, and who showed higher levels of physical activity and better adherence to the Mediterranean diet. We observed no relevant differences in age or body mass index according to energy-adjusted consumption of seafood products or LC ω-3 PUFA.

The association between energy-adjusted quintiles of consumption of different types of seafood products and depression is shown in Table 2. Total seafood consumption was not associated with depression. When we analyzed separately different types of seafood consumption, we observed a non-linear inverse association (*p* for trend = 0.759) between intermediate categories of consumption of fatty fish (approximately 10–25 g/day) and the prevalence of depression, suggesting a U-shaped relationship. As compared with the reference category, the ORs (95% CI) for the consecutive quintiles of consumption of fatty fish were 0.77 (0.63–0.94), 0.71 (0.58–0.87), 0.78 (0.64–0.96), and 0.84 (0.69–1.03), respectively. Similarly, moderate consumption of lean fish (approximately 20 g/day) was also associated with lower depression prevalence, with an OR for the 3rd vs. the 1st quintile of 0.77 (0.63–0.94).

Table 3 shows the association between energy-adjusted total ω-3, LC ω-3 PUFA, and ALA intake (in quintiles) and depression. As in the case of fatty fish consumption, moderate LC ω-3 PUFA intake was inversely associated with depression. The association was higher when EPA, DHA, and DPA were simultaneously analyzed. After adjusting for several potential confounding factors including the adherence to the Mediterranean diet, the OR for the 2nd, 3rd, and 4th quintiles of intake of total LC ω-3 PUFA were 0.75 (0.62–0.92), 0.77 (0.63–0.94), and 0.76 (0.62–0.92), respectively. The highest intake of total LC ω-3 PUFA was not significantly associated with lower prevalence of depression (OR = 0.83; 95% CI = 0.68–1.01). We did not find any significant association between ALA intake and depression.

In an ancillary analysis, we excluded from the analyses all depression cases in which age of depression diagnosis was not available (*n* = 25) or in which the diagnosis date was very remote (more than ten years before enrolment, *n* = 952). In this sub-sample (*n* = 5610, cases = 390) the results were no longer significant although the magnitude of effect was quite similar to that observed in the overall sample; the ORs and 95% CI for successive quintiles of fatty fish consumption were: 1 (ref.), 0.85 (0.60–1.20), 0.86 (0.61–1.20), 0.89 (0.63–1.25), and 0.90 (0.64–1.26). In the case of LC ω-3 PUFA, we found the following estimates for the association between quintiles of intake and depression: 1 (ref.), 0.83 (0.60–1.15), 0.83 (0.59–1.15), 0.71 (0.51–1.00), and 0.82 (0.59–1.13).

**Figure 1:**
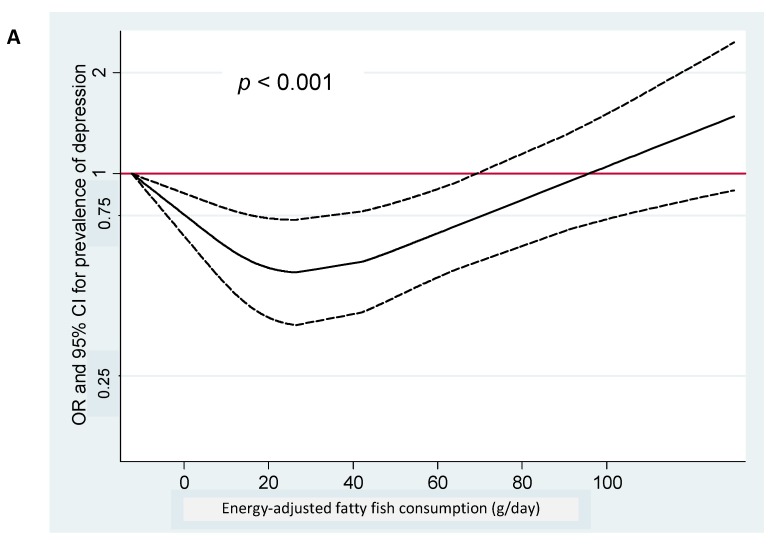

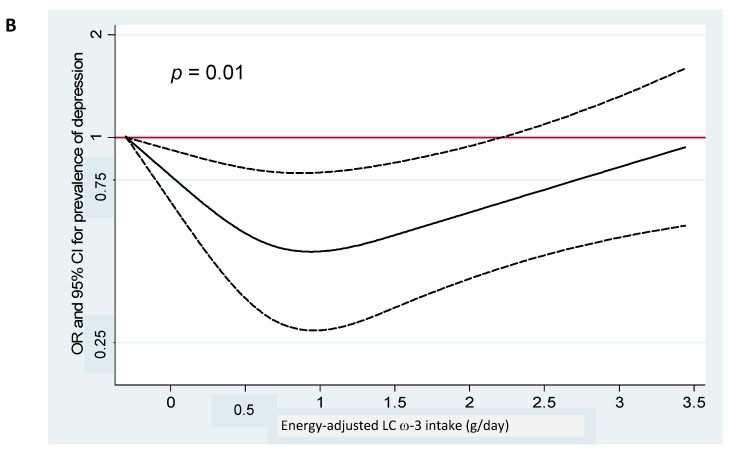
Spline regression models. (**A**) Spline regression model of the odds ratio of depression according to fatty fish consumption (the dotted lines represent 95% CI). (**B**) Spline regression model of the odds ratio of depression according to LC ω-3 PUFA intake (the dotted lines represent 95% CI).

To account for non-linear associations, we used restricted cubic spline analysis. We found a suggestion of U-shaped associations between fatty fish consumption or LC ω-3 intake and prevalent depression (Figure 1) as suggested in logistic regression analyses. Moderate intake (around 20–30 g/day of fatty fish or 0.5–1.5 g/day of LC ω-3) was inversely associated with depression prevalence.

The association between fish consumption and LC ω-3 PUFA intake and depressive symptoms, as appraised by the Beck Depression Inventory-II is shown in Table 4. Data were available only for 6562 participants, the 4 upper quintile values of fish/seafood consumption or LC w-3 PUFA intake were introduced as dummy independent variables. Participants with the lowest consumption of fatty and lean fish had the highest scores in the Beck Depression Inventory-II (a higher score meaning a higher probability of depression). Participants with a moderate consumption of both types of fish (3rd quintile) showed approximately 1 point less in the score of depressive symptoms than those with the lowest consumption (1st quintile). The regression coefficients (for the comparison between the 3rd vs. 1st quintiles) were −0.938 (−1.494 to −0.382) for fatty fish and −0.934 (−1.524 to −0.344) for lean fish (Table 4). Similar results were found for total LC ω-3 PUFA intake.

Table 5 shows the association between fatty fish consumption and LC ω-3 PUFA intake and depression prevalence according to different characteristics of the sample. In this subgroup analysis, we did not find any significant multiplicative interaction (effect modification) by any of these characteristics on the association between fatty fish consumption or LC ω-3 PUFA intake and depression. Although the inverse association between fatty fish consumption and depression seemed to be stronger among women and among participants with a lower adherence to the Mediterranean diet (MDS < 4 points), the interactions were not statistically significant (*p* for interaction = 0.558 and 0.437, respectively). Similarly, none of the product terms was statistically significant in the analysis of LC ω-3 PUFA.

## 4. Discussion

In this cross-sectional analysis of the PREDIMED-Plus trial, we observed a non-linear inverse association only for between moderate levels of fish consumption (particularly fatty fish consumption) and LC ω-3 PUFA intake and life-time prevalence of depression and depressive symptoms intensity, but not for the highest levels of intake (U-shaped relationship). The results were not modified by sex, by the presence of type 2 diabetes, or by several life-style factors such as smoking.

Although a recent study has found a dose–response inverse relationship between fish consumption and depression with an OR of 0.52 (95% CI: 0.37–0.74) for those participants with the highest consumption of fish (≥4 times/week) as compared to those with a consumption lower than 1 time/week [16], a meta-analysis for the same authors has failed to find a significant dose–response relationship between fish consumption and depression [11]. Our results are in accordance with this meta-analysis and with other studies that have found an inverse association between moderate fish consumption and mental disorders but suggesting a J-shaped or U-shaped relationship between fish consumption and LC ω-3 PUFA intake and depression. Several years ago, in another Spanish project, the SUN (Seguimiento Universidad de Navarra) cohort study, those participants with a moderate consumption of fish (third and fourth quintiles of consumption) had a relative risk reduction of mental disorders (that included not only depression but also anxiety and stress) higher than 30% [28]. More recently, Matsuoka et al. have also found this J-shaped association of intake of fish, EPA, or DPA with major depressive disorder after a long follow-up period [29]. This conclusion was also raised by a meta-analysis published in 2016 in which ω-3 PUFA decreased the risk of depression with an intake of up to 1.8 g/day [10]. A cross-national ecological study reported that a threshold of 750 mg/day of LC ω-3 PUFA intake was sufficient for protecting >98% of the populations worldwide from depression [30].

Several possible explanations may contribute to a better understanding of this non-linear association between fish consumption and depression. One of the more feasible explanations is the intake of other nutrients that could counteract the effect of fish or LC ω-3 PUFA intake on depression, including ω-6 PUFA intake as some authors have suggested [30,31]. Actually, ω-6 PUFA intake gradually increased with the increment in fish consumption. An alternative explanation for this plateau is that a threshold effect may exist, and once the threshold is achieved the inverse association with subsequent higher fish or LC ω-3 PUFA intake plateaued. Finally, we cannot exclude the possibility of reverse causality. Several participants with depressive symptoms (and a possible sub-clinical depression) might increase their fish and ω-3 PUFA intake in order to improve their symptoms. 

The results observed for specifically fatty and lean fish were not reproduced for other kinds of seafood products or for canned fish. The content in LC ω-3 PUFA could differ regarding fish/seafood sources, being higher in fatty fish and lower in other sea products such as mollusks, crayfish, or octopus. Although the level of LC ω-3 PUFA in canned fish (mainly tuna and anchovy in Spain) seems to be similar to that found in fresh fish [32], the consumption of this kind of product was very low in our cohort (the median intake in the highest quintile was only 22 g/day). Moreover, according to Czerner et al., the use of vegetable oil as covering liquid in some canned fish could lead to final products with increased ω-6 PUFA content [32]. These facts could explain the lack of association of this sea product with depression in our study.

The role of LC ω-3 PUFA in depression has been extensively evaluated as coadjuvant in antidepressant treatment [8,9]. The results regarding the role of ω-3 PUFA in depression prevention are less conclusive. Whereas some authors have reported a reduction in depression risk associated with its intake, others failed to observe any significant association [11]. Moreover, the results differ according to the type of ω-3 PUFA analyzed: ALA, EPA, or DHA [33,34]. One possible explanation for these differences is the differential anti-inflammatory role of the ω-3 PUFA. For example, DHA is prone to peroxidation leading to a less effective anti-inflammatory action than dietary EPA [35].

A large number of studies have reported the possible role of inflammation in depression through mechanisms such as activation of the hypothalamic–pituitary–adrenal axis, tryptophan depletion, neurotransmitter transport and metabolism disturbances, and decrease in brain-derived neurotrophic factor availability [5,36]. All these pathological mechanisms would explain the inverse association with depression found for LC ω-3 PUFA in our study.

The strengths of this study are its large sample size, the exposed populations consisting of people of both sexes, the adjustment for a wide array of potential confounders, and the use of validated tools to assess information. Some potential limitations of our study also need to be mentioned. The cross-sectional design of the study does not afford us the possibility of establishing any causal association between fish consumption and depression, and the presence of a possible reverse causality bias cannot be excluded. In fact, the presence of a depressive disorder could lead to less healthy dietary habits including lower fish consumption. Contrarily, participants with depression could also increase their fish or ω-3 PUFA intake to improve their depressive condition. Another possible caveat might be that our participants are not representative of the general Spanish population. Our participants were aged between 55 and 75 years, were overweight or obese, and all met criteria for metabolic syndrome. Nevertheless, the lack of representativeness does not preclude the establishment of associations [37]. Self-reporting of a clinical diagnosis or the use of medication was used as the criteria to establish depression with a potential presence of a misclassification bias. However, self-reported diagnosis of depression collected through a questionnaire seems to be a valid tool to ascertain depression according to a validation study carried out in a subsample of a Spanish cohort [22]. Similarly, although the validity of the semi-quantitative food-frequency questionnaire has been evaluated [19], some degree of misclassification may exist in the dietary assessment. Moreover, as our sample was composed of overweight/obese patients, our participants might tend to underreport their fish or ω-3 PUFA intake as some studies suggest [38]. However, this misclassification is more likely to be non-differential, and therefore would bias the results towards the null. Another fact that should be taken into account is the biological condition of our participants who are overweight and with metabolic syndrome. This condition could also modify fatty acid absorption and metabolism and thus, serum levels of ω-3 PUFA could substantially differ from dietary intake of this type of fats. Finally, although all the results were adjusted for a variety of major potential confounders (including adherence to the Mediterranean diet, smoking, or physical activity), we cannot exclude the presence of some unknown or unmeasured factors that could partly explain the reported results; the associations between fish consumption and depression might be due to confounding by healthier diet and lifestyle and better psychosocial circumstances.

In conclusion, the findings from the current study support the idea that moderate intake of fish and LC ω-3 PUFA (U-shaped relationship) may protect against depression independently of the sex differences, the presence of cardiometabolic disturbances, or life-style habits. More studies with longitudinal design are needed to confirm the reported results and definitively establish the role of seafood products and ω-3 PUFA in depression development.

## Figures and Tables

**Table 1:** Baseline characteristics of the participants from the PREDIMED-PLUS trial according to quintiles of consumption of fish/seafood and long-chain omega-3 fatty acids.

	Total Fish/Seafood				LC ω-3 PUFA (EPA + DHA + DPA)			
	Q1	Q3	Q5	*p*	Q1	Q3	Q5	*p*
Age (mean, SD)	64.3 (5.1)	65.3 (4.9)	65.1 (4.6)	<0.001	64.6 (5.1)	65.0 (5.0)	65.3 (4.7)	0.005
Sex (%)				<0.001				<0.001
Men	65.3	51.8	41.5		63.3	51.4	44.4	
Educational level				0.206				0.438
Primary	21.6	21.5	24.0		20.7	21.3	24.7	
Secondary	28.6	30.1	29.3		28.9	30.3	26.7	
University	49.2	47.3	46.1		49.8	47.6	47.7	
Unknown	0.6	1.2	0.5		0.6	0.8	0.9	
Marital status				0.723				0.647
Married	75.0	77.9	76.7		76.5	77.1	76.4	
Body mass index (mean, SD)	32.7 (3.4)	32.5 (3.4)	32.7 (3.4)	0.439	32.7 (3.4)	32.8 (3.4)	32.6 (3.4)	0.420
Smoking				<0.001				<0.001
Current smoker	15.9	13.2	9.9		14.5	12.6	9.9	
Ex-smoker	46.5	41.5	43.2		46.5	42.8	42.7	
Never smoker	37.6	45.3	46.8		39.0	44.6	47.4	
Physical activity (METs-min/w) (mean, SD)	2458 (2540)	2410 (2314)	2718 (2359)	0.010	2388 (2501)	2423 (2188)	2714 (2346)	0.002
Presence of diseases								
Hypercholesterolemia	66.7	69.4	69.5	0.044	66.8	69.4	70.0	0.002
Type 2 Diabetes	25.3	27.5	29.2	0.268	25.9	27.4	28.1	0.578
Hypertension	83.8	82.8	83.1	0.663	83.0	84.8	81.8	0.228
Mediterranean diet (0–8 score) (mean, SD)	3.6 (1.5)	3.9 (1.5)	4.0 (1.5)	<0.001	3.7 (1.5)	3.8 (1.5)	4.0 (1.5)	<0.001
Energy intake (Kcal/day) (mean, SD)	2476 (604)	2332 (535)	2356 (517)	0.010	2540 (593)	2219 (488)	2356 (486)	<0.001

SD: Standard Deviation; MET: Metabolic equivalent; LC ω-3 PUFA: Long-chain omega-3 polyunsaturated fatty acids; EPA: Eicosapentaenoic acid; DHA: Docosahexaenoic acid; DPA: Docosapentaenoic acid; Q1–Q5: Quintile 1–Quintile 5.

**Table 2:** Odds ratio (OR) and 95% confidence interval (CI) for the association between energy-adjusted fish/seafood consumption (quintiles) and life-time prevalence of depression in the PREDIMED-PLUS trial.

**Total Fish/Seafood**						
	**Q1**	**Q2**	**Q3**	**Q4**	**Q5**	***p* for trend**
Median (g/day)	40.53	67.95	90.70	117.26	155.28	
Cases	265	263	261	283	295	
Model 1	1 (ref.)	0.85 (0.70–1.03)	0.84 (0.69–1.02)	0.87 (0.72–1.06)	0.87 (0.71–1.05)	0.297
Model 2	1 (ref.)	0.87 (0.71–1.06)	0.86 (0.71–1.05)	0.91 (0.75–1.11)	0.93 (0.77–1.13)	0.777
Model 3	1 (ref.)	0.87 (0.71–1.06)	0.87 (0.71–1.06)	0.91 (0.75–1.11)	0.93 (0.76–1.13)	0.752
Model 4	1 (ref.)	0.86 (0.71–1.05)	0.86 (0.71–1.06)	0.91 (0.74–1.11)	0.94 (0.77–1.14)	0.851
	**Fatty Fish**					
	**Q1**	**Q2**	**Q3**	**Q4**	**Q5**	***p* for trend**
Median (g/day)	2.70	10.01	15.60	20.39	53.24	
Cases	277	259	231	301	299	
Model 1	1 (ref.)	0.78 (0.64–0.95)	0.69 (0.57–0.85)	0.80 (0.66–0.97)	0.80 (0.66–0.97)	0.338
Model 2	1 (ref.)	0.80 (0.65–0.97)	0.72 (0.59–0.88)	0.82 (0.68–1.00)	0.85 (0.70–1.03)	0.701
Model 3	1 (ref.)	0.79 (0.65–0.97)	0.72 (0.59–0.88)	0.82 (0.67–1.00)	0.85 (0.70–1.04)	0.711
Model 4	1 (ref.)	0.77 (0.63–0.94)	0.71 (0.58–0.87)	0.78 (0.64–0.96)	0.84 (0.69–1.03)	0.759
	**Lean Fish (excluding salty cod)**					
	**Q1**	**Q2**	**Q3**	**Q4**	**Q5**	***p* for trend**
Median (g/day)	5.87	15.33	21.54	57.80	63.43	
Cases	269	195	322	185	396	
Model 1	1 (ref.)	0.83 (0.68–1.03)	0.76 (0.62–0.92)	0.75 (0.60–0.92)	0.87 (0.71–1.06)	0.310
Model 2	1 (ref.)	0.86 (0.70–1.06)	0.78 (0.64–0.95)	0.79 (0.64–0.98)	0.91 (0.74–1.11)	0.614
Model 3	1 (ref.)	0.86 (0.70–1.06)	0.79 (0.64–0.96)	0.80 (0.65–0.99)	0.91 (0.75–1.11)	0.644
Model 4	1 (ref.)	0.86 (0.69–1.06)	0.77 (0.63–0.94)	0.83 (0.67–1.02)	0.88 (0.72–1.08)	0.658
	**Canned Fish (natural and oily)**					
	**Q1**	**Q2**	**Q3**	**Q4**	**Q5**	***p* for trend**
Median (g/day)	1.55	4.47	6.91	14.08	21.99	
Cases	260	269	266	299	273	
Model 1	1 (ref.)	1.00 (0.82–1.22)	0.95 (0.78–1.15)	1.17 (0.96–1.42)	1.02 (0.84–1.24)	0.356
Model 2	1 (ref.)	1.01 (0.82–1.23)	0.95 (0.78–1.16)	1.16 (0.96–1.41)	1.00 (0.82–1.22)	0.478
Model 3	1 (ref.)	1.01 (0.83–1.24)	0.95 (0.78–1.16)	1.16 (0.95–1.41)	1.00 (0.83–1.22)	0.509
Model 4	1 (ref.)	0.97 (0.80–1.19)	0.91 (0.75–1.12)	1.13 (0.93–1.38)	0.97 (0.79–1.18)	0.625
	**Other Seafood**					
	**Q1**	**Q2**	**Q3**	**Q4**	**Q5**	***p* for trend**
Median (g/day)	2.99	17.42	27.40	32.38	48.96	
Cases	295	262	265	279	266	
Model 1	1 (ref.)	0.89 (0.73–1.08)	0.89 (0.73–1.07)	0.93 (0.77–1.12)	0.89 (0.74–1.08)	0.328
Model 2	1 (ref.)	0.92 (0.76–1.12)	0.92 (0.76–1.12)	0.99 (0.82–1.20)	0.94 (0.77–1.14)	0.665
Model 3	1 (ref.)	0.91 (0.75–1.11)	0.91 (0.75–1.11)	0.99 (0.82–1.20)	0.93 (0.77–1.13)	0.622
Model 4	1 (ref.)	0.90 (0.74–1.10)	0.94 (0.77–1.14)	0.97 (0.80–1.18)	0.94 (0.77–1.14)	0.674

Q1–Q5: Quintile 1–Quintile 5, Model 1: Adjusted for sex and age; Model 2: Additionally adjusted for marital status, educational level, smoking, and physical activity; Model 3: Additionally adjusted for body mass index, hypercholesterolemia, hypertension, and type 2 diabetes mellitus; Model 4: Additionally adjusted for energy intake and adherence to the Mediterranean diet.

**Table 3:** OR and 95% CI for the association between energy-adjusted omega-3 fatty acid intake (quintiles) and life-time prevalence of depression in the PREDIMED-Plus trial.

**Total ω-3 PUFA (LC ω-3 PUFA + ALA)**						
	**Q1**	**Q2**	**Q3**	**Q4**	**Q5**	***p* for trend**
Median (g/day)	1.305	1.731	2.072	2.540	3.316	
Cases	265	281	269	282	270	
Model 1	1 (ref.)	0.96 (0.79–1.16)	0.88 (0.72–1.07)	0.91 (0.75–1.10)	0.85 (0.70–1.04)	0.107
Model 2	1 (ref.)	0.97 (0.79–1.17)	0.91 (0.75–1.11)	0.94 (0.78–1.15)	0.90 (0.74–1.10)	0.334
Model 3	1 (ref.)	0.96 (0.79–1.17)	0.92 (0.75–1.12)	0.94 (0.77–1.15)	0.91 (0.74–1.10)	0.362
Model 4	1 (ref.)	0.92 (0.75–1.12)	0.87 (0.71–1.06)	0.92 (0.75–1.12)	0.89 (0.73–1.08)	0.368
	**LC ω-3 PUFA (EPA + DHA + DPA)**					
	**Q1**	**Q2**	**Q3**	**Q4**	**Q5**	***p* for trend**
Median (g/day)	0.355	0.562	0.710	0.947	1.550	
Cases	286	255	266	274	286	
Model 1	1 (ref.)	0.76 (0.62–0.92)	0.78 (0.64–0.95)	0.75 (0.62–0.91)	0.79 (0.65–0.96)	0.126
Model 2	1 (ref.)	0.76 (0.66–0.93)	0.80 (0.66–0.97)	0.78 (0.64–0.95)	0.84 (0.69–1.01)	0.364
Model 3	1 (ref.)	0.77 (0.63–0.93)	0.80 (0.66–0.97)	0.77 (0.64–0.94)	0.84 (0.69–1.02)	0.355
Model 4	1 (ref.)	0.75 (0.62–0.92)	0.77 (0.63–0.94)	0.76 (0.62–0.92)	0.83 (0.68–1.01)	0.384
	**EPA + DHA**					
	**Q1**	**Q2**	**Q3**	**Q4**	**Q5**	***p* for trend**
Median (g/day)	0.235	0.407	0.535	0.754	1.167	
Cases	270	247	284	274	292	
Model 1	1 (ref.)	0.77 (0.63–0.94)	0.84 (0.69–1.02)	0.78 (0.64–0.95)	0.82 (0.67–0.99)	0.179
Model 2	1 (ref.)	0.78 (0.64–0.95)	0.87 (0.71–1.06)	0.81 (0.66–0.98)	0.87 (0.71–1.05)	0.462
Model 3	1 (ref.)	0.78 (0.64–0.95)	0.87 (0.72–1.06)	0.80 (0.66–0.98)	0.87 (0.71–1.05)	0.451
Model 4	1 (ref.)	0.77 (0.63–0.94)	0.85 (0.70–1.03)	0.79 (0.65–0.97)	0.87 (0.71–1.06)	0.538
	**α-Linolenic Acid (ALA)**					
	**Q1**	**Q2**	**Q3**	**Q4**	**Q5**	***p* for trend**
Median (g/day)	0.785	1.034	1.215	1.484	2.283	
Cases	266	286	275	279	261	
Model 1	1 (ref.)	1.00 (0.82–1.21)	0.95 (0.78–1.15)	0.98 (0.81–1.20)	0.87 (0.72–1.06)	0.144
Model 2	1 (ref.)	1.01 (0.83–1.22)	0.95 (0.78–1.16)	1.01 (0.83–1.22)	0.91 (0.75–1.11)	0.319
Model 3	1 (ref.)	1.02 (0.84–1.23)	0.96 (0.79–1.17)	1.02 (0.83–1.24)	0.92 (0.76–1.13)	0.398
Model 4	1 (ref.)	0.95 (0.78–1.16)	0.87 (0.71–1.08)	0.95 (0.77–1.17)	0.89 (0.72–1.09)	0.346

Q1–Q5: Quintile 1–Quintile 5; LCω-3 PUFA: Long-chain omega-3 polyunsaturated fatty acids; EPA: Eicosapentaenoic acid; DHA: Docosahexaenoic acid; DPA: Docosapentaenoic acid; Model 1: Adjusted for sex and age; Model 2: Additionally adjusted for marital status, educational level, smoking, and physical activity; Model 3: Additionally adjusted for body mass index, hypercholesterolemia, hypertension, and type 2 diabetes mellitus; Model 4: Additionally adjusted for energy intake and adherence to the Mediterranean diet.

**Table 4:** Scores in the Beck Depression Inventory-II according to quintiles of consumption of fish/seafood or long-chain omega-3 fatty acids in the PREDIMED-Plus trial.

	Regression Coefficient *	95% Confidence Interval
**Total seafood**		
Q1	0 (ref.)	
Q2	−0.692	−1.242 to −0.141
Q3	−0.595	−0.648 to 0.459
Q4	−0.571	−1.127 to −0.015
Q5	−0.478	−1.040 to 0.083
**Fatty fish**		
Q1	0 (ref.)	
Q2	−0.715	−1.268 to −0.163
Q3	−0.938	−1.494 to −0.382
Q4	−0.719	−1.290 to −0.149
Q5	−0.504	−1.071 to 0.064
**Lean fish**		
Q1	0 (ref.)	
Q2	−0.528	−1.082 to 0.025
Q3	−0.934	−1.524 to −0.344
Q4	−0.609	−1.164 to −0.055
Q5	−0.844	−1.473 to −0.216
**LC ω-3 PUFA (EPA + DHA + DPA)**		
Q1	0 (ref.)	
Q2	−0.487	−1.038 to 0.064
Q3	−0.923	−1.481 to −0.364
Q4	−0.413	−0.970 to 0.144
Q5	−0.428	−0.987 to 0.130
**EPA + DHA**		
Q1	0 (ref.)	
Q2	−0.720	−1.273 to −0.167
Q3	−0.777	−1.340 to −0.213
Q4	−0.398	−0.958 to 0.161
Q5	−0.533	−1.097 to 0.031

LC ω-3 PUFA: Long-chain omega-3 polyunsaturated fatty acids; EPA: Eicosapentaenoic acid; DHA: Docosahexaenoic acid; DPA: Docosapentaenoic acid. * Model adjusted for sex, age, marital status, educational level, smoking, physical activity, body mass index, prevalence of hypercholesterolemia, hypertension, diabetes, energy intake, and adherence to the Mediterranean diet.

**Table 5:** OR * and 95% CI for the association between energy-adjusted fatty fish consumption and LC ω-3 PUFA intake (quintiles) and life-time prevalence of depression in the PREDIMED-Plus trial according to different characteristics of the sample.

	**Fatty Fish**				
	**Q2**	**Q3**	**Q4**	**Q5**	***p* interaction**
Sex					0.558
Men (3393)	0.78 (0.57–1.06)	0.71 (0.52–0.98)	0.95 (0.69–1.32)	0.85 (0.61–1.20)	
Women (3194)	0.74 (0.57–0.97)	0.71 (0.54–0.94)	0.70 (0.54–0.91)	0.82 (0.63–1.06)	
Diabetes **					0.932
No (4759)	0.74 (0.59–0.94)	0.69 (0.54–0.88)	0.76 (0.60–0.96)	0.81 (0.64–1.03)	
Yes (1810)	0.82 (0.56–1.21)	0.73 (0.49–1.10)	0.81 (0.55–1.20)	0.87 (0.59–1.28)	
Mediterranean diet					0.437
<4 (2717)	0.73 (0.55–0.96)	0.65 (0.48–0.88)	0.64 (0.48–0.85)	0.82 (0.61–1.11)	
≥4 (3870)	0.82 (0.61–1.09)	0.78 (0.59–1.04)	0.93 (0.70–1.24)	0.88 (0.67–1.16)	
Smoking					0.588
Non-smoker (5771)	0.74 (0.60–0.92)	0.70 (0.57–0.87)	0.75 (0.61–0.93)	0.84 (0.68–1.04)	
Current smoker (816)	0.93 (0.54–1.60)	0.71 (0.39–1.28)	1.02 (0.55–1.88)	0.74 (0.40–1.39)	
	**LC ω-3 PUFA (EPA + DHA + DPA)**				
	**Q2**	**Q3**	**Q4**	**Q5**	***p* interaction**
Sex					0.951
Men (3393)	0.73 (0.54–1.00)	0.74 (0.54–1.02)	0.78 (0.56–1.08)	0.77 (0.55–1.07)	
Women (3194)	0.77 (0.60–1.00)	0.79 (0.61–1.03)	0.75 (0.58–0.97)	0.87 (0.67–1.11)	
Diabetes **					0.951
No (4759)	0.73 (0.58–0.92)	0.75 (0.60–0.95)	0.77 (0.61–0.97)	0.82 (0.65–1.04)	
Yes (1810)	0.82 (0.56–1.22)	0.81 (0.55–1.20)	0.73 (0.49–1.07)	0.83 (0.56–1.21)	
Mediterranean diet					0.821
<4 (2717)	0.68 (0.51–0.91)	0.71 (0.53–0.94)	0.69 (0.52–0.92)	0.81 (0.60–1.09)	
≥4 (3870)	0.83 (0.63–1.09)	0.84 (0.64–1.12)	0.81 (0.62–1.06)	0.87 (0.67–1.13)	
Smoking					0.234
Non-smoker (5771)	0.71 (0.57–0.88)	0.78 (0.63–0.97)	0.75 (0.61–0.93)	0.85 (0.69–1.05)	
Current smoker (816)	1.06 (0.62–1.81)	0.61 (0.34–1.11)	0.78 (0.44–1.41)	0.59 (0.31–1.12)	

* Model adjusted for sex, age, marital status, educational level, smoking, physical activity, body mass index, prevalence of hypercholesterolemia, hypertension, diabetes, energy intake, and adherence to the Mediterranean diet. ** 18 missing data regarding diabetes status.
